# Translation and adaptation of the ***Competencias Esenciales en Salud Pública para los recursos humanos en salud*** **^1^**

**DOI:** 10.1590/1518-8345.1684.2896

**Published:** 2017-06-05

**Authors:** Maria de Lourdes de Almeida, Aida Maris Peres, Maria Manuela Frederico Ferreira, Maria de Fátima Mantovani

**Affiliations:** 2 Doctoral student, Universidade Federal do Paraná, Curitiba, PR, Brasil. Scholarship holder at Coordenação de Aperfeiçoamento de Pessoal de Nível Superior (CAPES), Brazil. Professor, Universidade Estadual do Oeste do Paraná, Foz do Iguaçu, PR, Brazil.; 3 PhD, Associate Professor, Universidade Federal do Paraná, Curitiba, PR, Brazil.; 4 PhD, Professor Coordinator, Escola Superior de Enfermagem de Coimbra, Coimbra, Portugal.

**Keywords:** Translating, Professional Competence, Public Health, Health Management, Health Personnel Management

## Abstract

**Objective:**

to perform the translation and cultural adaptation of the document named *Marco Regional de Competencias Esenciales en Salud Pública para los Recursos Humanos en Salud de la Región de las Américas* (Regional Framework of Core Competencies in Public Health for Health Human Resources in the Region of Americas) from Spanish to Brazilian Portuguese.

**Method:**

a methodological study comprising the following phases: authorization for translation; initial translation; synthesis of translations and consensus; back-translation and formation of an expert committee.

**Result:**

in the translation of domain names, there was no difference in 66.7% (N = 4); in the translation of domain description and competencies there were divergences in 100% of them (N = 6, N = 56). A consensus of more than 80% was obtained in the translation and improvement in the expert committee by the change of words and expressions for approximation of meanings to the Brazilian context.

**Conclusion:**

the translated and adapted document has the potential of application in research, and use in the practice of collective/public health care in Brazil.

## Introduction

The essential competencies in public health are defined by the knowledge, skills and attitudes for professional practice in public health care^1^ ^-^ ^2^ that are relevant for decision-making and resolution of population's health problems effectively and efficiently. They refer to the competencies all health professionals working in public health must possess, regardless of the practice scenario in which they develop their activities^2^ ^-^ ^3^.

The Regional Core Competency Framework for Public Health (MRCESP - Marco Regional de Competências Essenciais em Saúde Pública) can be an instrument to characterize public health knowledge, skills and attitudes. The document is a response to the challenges identified by the region of Americas to facilitate training of excellence, collaborate and give consistency to the public/collective health workforce of health systems^2^ ^-^ ^3^. The fundamental strategy of this proposal is based on the following fundamental axes: construction of the MRCESP and measurement of competencies; planning of regional and national capacitation, among other initiatives such as the virtual field of public health^2^ ^-^ ^3^. The MRCESP complements other core strategies of the Pan American Health Organization (PAHO), the World Health Organization and the United Nations, such as Primary Health Care, Essential Public Health Functions (EPHF), and the Development Goals of the Millennium^2^ ^-^ ^3^.

The construction of the MRCESP was based on the meetings organized between 2005 and 2008 by the PAHO's Human Resources for Health Project. Each country was designated for the elaboration of a domain. Brazil was responsible for the first domain, Peru for the second, Puerto Rico for the third, Colombia for the fourth, Chile for the fifth, and Mexico for the sixth domain. These meetings converged on the importance of training focused on development of human resources for public health in the Region of Americas for the consolidation of plans of training and continuous updating, and the need to define essential competencies with performance-oriented approach in health work^2^.

Thus, the MRCESP is a support proposal for countries strengthening their public health systems. It is constituted by 56 essential competencies distributed in six domains: nine competencies are of the *Análisis de situacion de salud* domain (Health situation analysis); 14 of the *Vigilancia y control de riesgos y danos* domain (Surveillance and control of risks and threats); ten competencies of the *Promoción de la salud y participación social* domain (Health promotion and social participation); seven of the *Políticas, planificación, regulación y control* domain (Policy, planning, regulation and control); Eight of the *Equidad en el accesso, y calidad en los servicios individuales y colectivos* domain (Equitable access, and quality of individual and public health services); and eight in the field of the *Salud internacional y salud global* (International and global health)^2^ ^-^ ^3^. These domains were elaborated from the EPHF, an instrument defined by PAHO that describes competency trends and the actions required by health systems in order to address the central objective of public health^2^ ^-^ ^3^.

In the search for professionals prepared for the predominant health care model reality in Brazil and its epidemiological context, the importance of studies in the area of development of health human resources stands out. They point to new work models based on new ways of management and teaching in order to adjust to changes in the health care model^4^. To this end, the adoption of the MRCESP reference may contribute to the construction of instruments that help in the development of professionals. This view justifies the translation and adaptation of this document to the Brazilian context.

Given the exposed, the objective of this study was to translate into Brazilian Portuguese and adapt to the Brazilian context the essential competencies for public health professionals suggested by the Pan American Health Organization, as described in the document *Marco Regional de competencias esenciales en salud pública para los recursos humanos en salud de la región de las Américas* (Regional Framework of Core Competencies in Public Health for Health Human Resources in the Region of Americas).

## Methodology

This is a methodological study for translation and cross-cultural adaptation of the essential public health competencies suggested by PAHO for human resources in health, which followed international recommendations^5^ ^-^ ^6^. Translation and cross-cultural adaptation followed the phases: 1. Authorization of the lead author and PAHO for translation; 2. Initial translation; 3. Synthesis of translations and consensus; 4. Back-translation; 5. Composition of an expert committee.

These recommendations^5^ ^-^ ^6^ are for translation and cultural adaptation of research instruments. In this article, the adopted procedures varied because the translation and cultural adaptation were based on a PAHO document and not on a research instrument. Therefore, the pre-test application phase was not performed for psychometric evaluation and the phase of content evaluation by the expert committee closed the process.

The process of translation and cross-cultural adaptation requires a method for achieving equivalence between the source language and the target language. For instruments and documents used by cultures other than that of the source language, the ideal is not only the language translation, but a cultural adaptation to maintain the instrument content validity in different cultures^7^ ^-^ ^8^. This situation demands planning before starting the process^8^.

This process of translation and adaptation was performed from March 2014 to March 2015 in the south of Brazil. The first phase involved requesting permission to translate and use the reference document named *Marco Regional de competencias esenciales en salud pública para los recursos humanos en salud de la región de las Américas* (Regional Framework of Core Competencies in Public Health for Health Human Resources in the Region of Americas) to its first author and to the Pan American Health Organization, the director institution of the collective construction of the document by specialists.

The second phase was the initial translation of the original Spanish version of the document into Portuguese. It was performed by three qualified independent translators, named T1, T2 and T3, who met the following inclusion criteria: proficiency in Spanish, experience in Spanish speaking countries, and domain of the Portuguese language. The initial contact with these translators was by e-mail and, after accepting to participate in the translation, an original version of the document was sent to them.

This phase involved participation of the three translators. The first translator (T1) was a nurse with stricto sensu training (equivalent to specialization), doctorate level in Public Health in a Spanish-speaking country, fluent in the language, with knowledge on the specific terms of the document, and aware of the study objectives. The second translator (T2) was a nurse born in Spain and fluent in Portuguese. The third translator (T3) was a Spanish language specialist, native of a Spanish-speaking country, and working on translation of materials from Spanish into Portuguese. The second and third translators were unaware of the study objectives in order to perceive interpretation errors and ambiguities in the versions translated by them.

The third phase of the study comprised the synthesis of translations and consensus. The researcher produced a synthesis of the three versions translated from Spanish to Portuguese, which were analyzed individually to identify differences between translations. The translated versions were forwarded to translators by email with divergences highlighted for comparisons between their translation and the other translated versions with the original document.

In the fourth phase, back translation or translation back into Spanish, the first translator (T1R) of the Portuguese version for Spanish was an expert in Spanish, professionally acting as a translator and interpreter of the language, and native of a Spanish speaking Latin American country. The second translator (T2R) had a degree in Languages (emphasis in Spanish), worked as a teacher and translator of the Spanish language, and was also native of a Spanish speaking Latin American country.

The original version of the document was compared to the back-translated version to check the trustworthiness of the document in translation and cultural adaptation. A web conference was held with this purpose, because a meeting face-to-face was difficult considering that translators were in different places from researchers. A new version in Spanish and the definitive version in Portuguese (P3) were obtained as a result of this phase.

The fifth phase comprised the cultural adaptation, content evaluation and analysis of cross-cultural equivalence. For the process, was formed a committee including two teaching nurses from the collective health area, a dentist and a physician acting in basic health care management in the studied municipality, and a teacher of the undergraduate course in languages (emphasis on Spanish), all of which with mastery of the Spanish language.

Members of the expert committee were selected according to the inclusion criteria: mastery of the Spanish language; active health professional working in teaching, research or practice of collective health management; or a professional of the studied language. The final version in Portuguese was defined in this phase based on the absence of divergences on cultural adaptation reported by the expert committee members.

The study was approved by the Committee of Ethics in Research with Human beings - (CEP - Comitê de Ética em Pesquisa) of a Brazilian public university under CAAE number 36031414.0.0000.0102 and number 832.463.

## Results

In the translation of the names of the six domains, there was no translation difference in 66.7% (N = 4), and in 33.3% (N = 2) there were divergences in domain one and domain four, which were resolved with discussion between translators and the researcher. Figure 2 presents the results of the translation and cultural adaptation of the name and description of each domain of the MRCESP after performing the five methodological phases.

**Figure 1:**
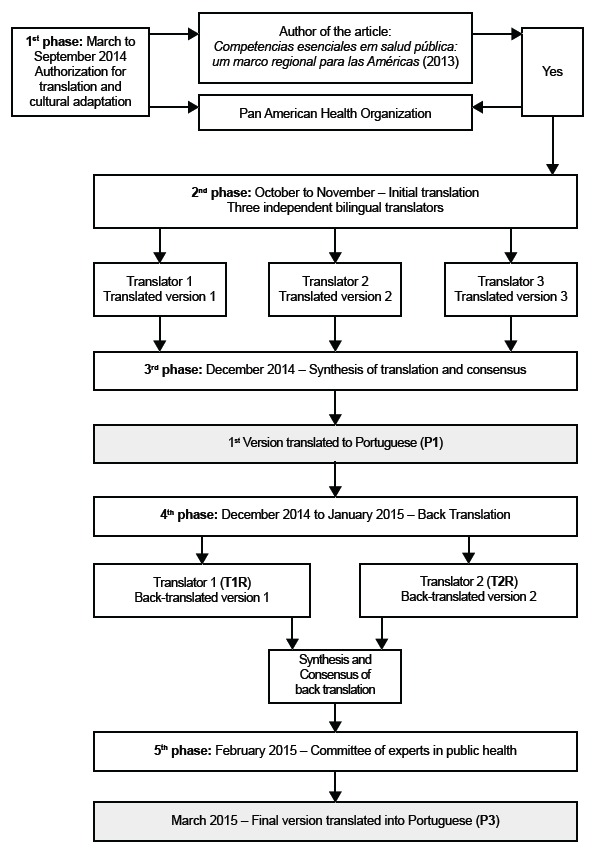
Schematic representation of the methodological procedure and chronology for the translation and cross-cultural adaptation of the *Marco Regional de competencias esenciales em salud publica para los recursos humanos em La salud de la region de las Américas* (Regional Framework of Core Competencies in Public Health for Health Human Resources in the Region of Americas).

**Figure 2:**
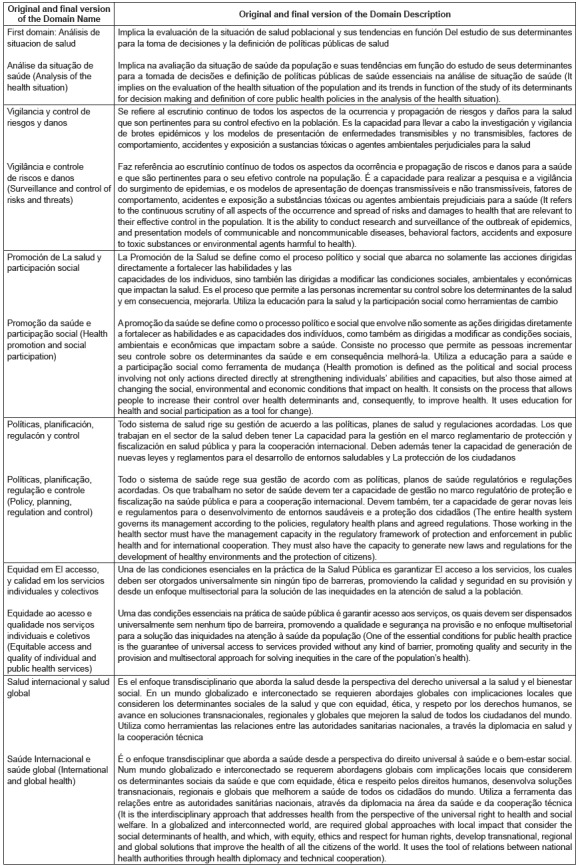
Translation and cultural adaptation of the name and description of domains of core competencies for human resources in public health. Brazil, 2016

In all domains, were found differences in the translation of the competency description, but the majority (above 80%) obtained consensus in this same round, and the same happened with the words pointed as divergent.

Terms or words with the same translation among translators were not presented at the consensus meeting. After reaching consensus about the terms with differences from the initial translation, was obtained the first version translated into Portuguese. This version was sent to the back-translation and there were no significant divergences between the translation of the back-translated version in Portuguese, and the document original version in Spanish. The first Portuguese version was submitted to the expert committee for cultural adaptation and analysis of semantic, cultural, idiomatic and conceptual equivalence of the translated document, which resulted in minor changes. Thus, after the committee of experts, was obtained the final version of the translated document.

## Discussion

Despite the suggestions of core competencies for Latin America, in the process of translation and adaptation to the Brazilian context it was more difficult to reach consensus in the initial translation performed by three independent translators. The expert committee acted to review the versions and carry out the cultural adaptation.

The first domain, Análise da Situação de Saúde (Health Situation Analysis), describes the competencies required from public health professionals for this analysis. It can be a support instrument for decision making and health action planning by setting priorities and building prospective scenarios that consider the evaluation of health actions, programs and policies^8^.

The competencies of the second domain, Vigilância e Controle de Riscos e Danos (Vigilance and Control of Risks and Threats), are important for public health professionals. Socio-environmental vulnerability is a result of social processes and environmental changes, a combination of difficulties in conditions of work, income, health and education, and also related to the lack of safe and healthy housing, sanitation, making certain population groups, the poorest in particular, vulnerable to disasters^9^.

In Brazil, there have been some achievements with health surveillance actions, namely the systematic and regular use of research on behavioral risk factors, such as Vigitel, to detect changes in prevalence rates of behaviors and health risks; the universal notification of significant events as the case of interpersonal violence; the use of administrative data such as emergency and hospital data and to track injuries; and the techniques of linkage, and capture and recapture for the evaluation of information system processes^10^.

The competencies of the third domain, Promoção da Saúde e Participação Social (Health Promotion and Social Participation), are strengthened with the consensus on the importance of inserting citizens in the production of care. Competent professionals in this scope can broaden their role by contributing assertively to the development of health system reforms in order to promote citizenship projects and participation in decision-making and delineation of collective goals^11^.

Health and education policies, when pointing guidelines for the training and performance of health professionals, take the promotion of health into consideration. This focus still persists in health services, although during operationalization and in response to demands there is greater concern with addressing the problem than with health promotion.

The fourth domain is called Políticas, Planejamento, Regulação e Controle (Policy, Planning, Regulation and Control). It requires that professionals have competencies focused on knowledge about the local health situation for implementing measures based on the population health needs. The use of rational planning in the epidemiological logic contributes to directing the formulation and evaluation of health policies^12^.

The fifth domain is entitled Equidade ao Acesso e Qualidade nos Serviços Individuais e Coletivos (Equitable Access and Quality of Individual and Public Health Services). The development and acquisition of its competencies is perceived as an impact factor to guarantee universal access to health that is achieved by removing barriers to the use of integral health services equitably. Therefore, human resources have a central role, despite the great difficulties still perceived on this theme in primary health care^13^.

The competencies of the sixth domain, Saúde Internacional e Global (International and Global Health) are as important as current, and in times of globalization, it is essential that public health professionals have such competencies. The term global health goes beyond the existing divide between the rich and poor, the developed and in development, and the national boundary lines^14^ ^-^ ^15^. This domain is focused on the cultural, social, economic and political possibilities outlining the world scope^14^.

For their solution, global health problems require international cooperation to address issues related to old causes of death in Brazil that are still priority problems in other countries, such as malnutrition and child mortality. In the present and future, collaboration between countries demands the joint overcoming of challenges to advance in the care for violence and chronic-degenerative diseases, such as cardiovascular disease, lung disease, cancer and diabetes. Global health also includes preventive issues addressed to different populations, and has the greater objective of equity and access to health care for all^15^.

Regarding the debate on the construction of competency models for public health, the movement of the Galway Consensus Conference took place in 2008. It showed an international concern with the theme in this area, facing a new health scenario and the demand for professionals prepared for it^4^. The aim of this event was the promotion of dialogue and knowledge exchange among international scholars on areas of competency, norms and ways of ensuring quality in the preparation of professionals and specialists in health education for the practice of health promotion^16^.

The identification of these competencies aims at the development of the workforce because it is a starting point and reinforcement for health organizations understanding the need for this type of investment for improving health care provision^1^. It is an institutional role to solve the population health problems efficiently and effectively, with the necessary requisites for all professionals who practice in public health, regardless of their field of activity or activity developed^2^.

More specifically, core competencies can be used for assessment of public health personnel performance in order to identify needs for training, development and planning for continuing education, and elaboration of job description. Therefore, organizations providing public health services can interpret and adapt core competencies for meeting specific organizational needs^2^.

## Final considerations

The document named *Marco Regional de Competencias Esenciales em Salud Pública para los Recursos Humanos en Salud de la Región de las Américas* (Regional Framework of Core Competencies in Public Health for Health Human Resources in the Region of Americas) was translated and adapted to the Brazilian context following international guidelines procedures. This study allows the use of skills translated and adapted to the Brazilian context as a reference for what is expected from human resources working in public health care in Brazil, because they define knowledge, skills and attitudes related to this area.

The potential for application and use of this translated document for knowledge production in the various dimensions of professional practice in collective/public health in Brazil, and to collaborate for the improvement of health care provided to the population are noteworthy. Future research on the use of this competency framework in collective/public health care is recommended.
